# Multimodal Test Item Parameter Prediction From Text, Images, and Metadata: Fusing Together AI Vision and Language Models

**DOI:** 10.1177/00131644261460779

**Published:** 2026-07-13

**Authors:** Hotaka Maeda, Yikai “EK” Lu

**Affiliations:** 1 Smarter Balanced, Santa Cruz, CA, USA; 2 University of California, Santa Cruz, USA; 3 University of Minnesota Twin Cities, Minneapolis, USA

**Keywords:** large-scale assessment, natural language processing, artificial intelligence, DeBERTaV3, DINOv3, question difficulty prediction, item response theory

## Abstract

We propose a flexible multimodal model for predicting all dichotomous and polytomous item parameters from text, images, and metadata by fusing representations from encoder Transformer vision and language models. This deep learning model accommodates heterogeneous item formats, including items with any number of components such as correct and incorrect options, stimuli, and images. Answer key indicators distinguish correct options from distractors, while an attention pooling technique weights the relative importance of these components. Based on the two-parameter logistic model and the generalized partial credit model, we predict all item parameters jointly using a masking strategy to ensure that only relevant parameters contribute to the loss. Item-level and item component-level metadata are also included. We evaluate the approach using 40,965 English language arts and mathematics items for Grades 3–11. A single model accommodated both exam subjects, all 11 item types, and all item parameters, eliminating the need for multiple specialized models. However, results indicate that the full model was unable to leverage all input data. Often, prediction accuracy was unchanged as features were removed. Images were good predictors on their own (item intercept $R^{2}=.25$ ) but did not consistently contribute unique information when combined with text and metadata. Of the strongest-performing models, the most parsimonious variant achieved $R^{2}$ values of .67, .45, .39, .34, .66, .33, and .74 for item intercept, discrimination, difficulty, and four polytomous step threshold parameters, respectively. Findings suggest that current training methods may limit the learnability of complex multimodal deep fusion models. Example Python code from this study is available on Github: https://github.com/hotakamaeda/multimodal_prediction

## Introduction

To ensure the quality of items on standardized tests before operational use, test developers examine item characteristics like difficulty and discrimination (Crocker & Algina, 1986). Item response theory (de Ayala, 2009) is widely used to model item characteristics. Predicting the parameters of these models has several important applications in the development of large-scale assessments, including evaluating items before field testing (S. Han, Rijmen, et al., 2025; M. Li et al., 2025; Maeda, 2025; Maeda & Lu, 2025; Scarlatos et al., 2025), improving parameter estimation by incorporating predicted values as a Bayesian prior (Ulitzsch et al., 2025), or even using predicted values to replace calibration, such as when automatically generating items Circi et al. (2023) on the fly during computerized adaptive testing Baylari and Montazer (2009). With the increasing demand of such applications, accurate item parameter prediction methods are becoming increasingly desirable.

Although successful difficulty prediction methods exist (see reviews AlKhuzaey et al., 2024; Benedetto et al., 2023; Peters et al., 2025), one limitation in the current literature is that they fall short of being able to incorporate diverse types and features of items commonly present in large-scale assessments. With a few exceptions (El Masri et al., 2017; S. Han, Rijmen, et al., 2025; Yi et al., 2024), most difficulty prediction papers have focused on just one item type, typically multiple-choice questions (Peters et al., 2025). However, in practice, a test might contain varied item types to measure multiple content standards. One challenge in accommodating different item types in a prediction model is that researchers have not reached a consensus on the best way to incorporate and distinguish the various text components in prediction, such as passages, prompts, distractors, and correct answers. Another challenge is that image data commonly found on test items (e.g., plots and figures) are difficult to model and thus have not been used for predicting item parameters, despite their influence on text comprehension (Schnotz, 2005), item difficulty (Wu et al., 2015), and learning (Mayer, 2002; Paivio, 1990).

Another limitation in the current literature is that only a limited set of item response theory models and parameters has been used for this task, with most studies relying primarily on dichotomous models. For example, to our knowledge, S. Han, Rijmen, et al. (2025) is the only paper that attempted to predict polytomous item threshold parameters. Furthermore, although item discrimination is a good indicator of item quality, only a limited number of studies have attempted to predict it (e.g., S. Han, Rijmen, et al., 2025; Hillsley, 2025; Maeda, 2025; Stanger-Hall, 2012). The few that did have consistently found that item type is a good predictor of item discrimination (Hillsley, 2025; Stanger-Hall, 2012).

To address these shortcomings, the purpose of this study is to build flexible multimodal models that can utilize all available components of the item, including the text, images, and metadata, to predict all parameters of dichotomous and polytomous items. Notably, the models are designed to handle various item types simultaneously, such as items that contain zero to any number of response options, passages, and images while being able to recognize the correct and incorrect response options. The design allows the model to adapt a wide variety of simple to highly complex assessment data.

## Related Works

### Transformer-Based Language Models

Traditionally, item parameter prediction involved expert judgment (e.g., Wauters et al., 2012), simple syntactic features like word count and term frequency (e.g., Benedetto et al., 2020), or semantic features like word embeddings (e.g., Hsu et al., 2018). However, more recently, modern approaches to predicting item parameters from text have shifted to leveraging transformer-based language models, which greatly enhanced prediction accuracy potential (e.g., S. Han, Rijmen, et al., 2025; M. Li et al., 2025; Maeda, 2025; Scarlatos et al., 2025). Introduced by Vaswani et al. (2017), the transformers fundamentally advanced natural language processing by replacing recurrent architectures with attention mechanisms that process all input tokens in parallel. This design enables efficient training and scalable modeling of long-range dependencies. Transformer-based language models such as Bidirectional Encoder Representations from Transformers (BERT; Devlin et al., 2019) are pretrained on large text corpora and can be fine-tuned for downstream tasks including classification, regression, and question answering. Text inputs are tokenized into embeddings, which are passed through multiple encoder layers to produce representations that capture contextual semantic relationships of words.

In this study, the DeBERTaV3-large model (He et al., 2021) was used. DeBERTa extends BERT (Devlin et al., 2019) and RoBERTa (Liu et al., 2019) by disentangling content and positional information into separate representations and attention mechanisms, improving modeling of word relationships. DeBERTaV3 further improves training efficiency through replaced token detection, resulting in a 304-million-parameter model that achieves strong benchmark performance.

### Images and Vision Models

Visual information plays a critical role in supporting comprehension and learning by complementing textual information. According to dual coding theory, learners process verbal and visual information independently, and integrating both can enhance understanding and recall (Paivio, 1990). Building on this framework, multimedia learning theory posits that well-aligned text and images facilitate deeper learning by helping students organize and integrate information more effectively (Mayer, 2002). Empirical research has shown that diagrams, illustrations, and graphs can improve comprehension, problem-solving, and inference-making, particularly when visual elements convey structural or relational information that is difficult to express in text alone (Schnotz, 2005). In assessment contexts, images may therefore influence how students interpret item prompts, reduce ambiguity, or support reasoning processes, potentially affecting perceived difficulty and response behavior. Conversely, poorly designed visuals may increase extraneous cognitive load, raising item difficulty or lowering item discrimination. Therefore, past research suggest that images may alter item functioning, providing a theoretical basis for incorporating visual representations when predicting item parameters.

Various types of vision models are available in image-understanding contexts. For example, optical character recognition (OCR) is an automated process of detecting and converting text contained in images or scanned documents (Smith, 2007). Alternatively, Donut (Kim et al., 2022) directly maps document images to structured text representations without OCR, making them effective for text extraction tasks. Vision-language models such as Bootstrapping Language-Image Pre-training (BLIP; J. Li et al., 2022) and BLIP-2 (J. Li, Li, et al., 2023) learn image–text representations and are commonly used for image captioning and visual question answering. Other approaches include Contrastive Language-Image Pre-training (CLIP), which aligns images and text in a shared embedding space through contrastive learning (Radford et al., 2021), as well as convolutional neural networks (CNNs) and hybrid CNN–vision Transformer architectures used for diagram and chart understanding (Khan et al., 2023).

While these models are effective for text-centric or generative tasks, they are often optimized for image-text alignment rather than learning image-specific visual representations. In this study, we instead use a general-purpose self-supervised vision Transformer named DINOv3 (Oquab et al., 2023; Siméoni et al., 2025). The input is a 224 × 224 pixel image and outputs a fixed-length visual feature vector that can be used for downstream tasks such as classification or regression. We chose to use this model for three reasons. First, DINOv3 is trained to learn visual representations directly from images, rather than translating visual content into textual form, which may avoid redundancy when paired with a dedicated language model. Second, transformer-based vision models are modern and provide efficient and scalable computation while capturing global spatial relationships. Third, DINOv3 produces flexible and transferable image embeddings that can generalize across the diverse diagrams, figures, and visual stimuli commonly found in assessment items. This design makes DINOv3 particularly well suited for multimodal item parameter prediction, where visual information contributes information beyond text alone.

### Answer Key Indicators

Prior research has not identified an elegant method for incorporating the response option text into item parameter prediction models. In many studies, correct options, distractors, or both are excluded or simply concatenated to the item prompt without an indication of the answer key (Peters et al., 2025). Including these options has had little to no change in predictive accuracy (e.g., Benedetto, 2023). Such models lack the information necessary to identify miskeyed items or items containing more than the intended number of correct answers.

This limitation is consequential, as miskeyed items typically exhibit negative discrimination parameters. Moreover, established item development guidelines emphasize that the number and quality of distractors affect difficulty and discrimination (Haladyna et al., 2002; Susanti et al., 2017). The distinction between correct and incorrect options therefore contains critical construct-relevant information, untapped in item parameter prediction.

To address this limitation, we propose a technique termed “answer key indicators.” Answer key indicators consist of a set of dummy variables that explicitly encode whether each response option text component is keyed as correct or incorrect. These indicators are incorporated alongside the response option text and are used within the attention pooling mechanism, allowing the model to differentially weight correct answers and distractors when generating representations for item parameter prediction.

### Attention Pooling

Items often have multiple components, such as passages, prompts (i.e., stem), options, and images. Each of these pieces may contain unique information valuable for predicting item parameters, which need to be combined during prediction. A simple approach would be to average the information among these components, named mean pooling. A more advanced method, called attention pooling, is a learnable weighted averaging mechanism that assigns higher weights to more predictive components (Er et al., 2016). Through attention pooling, deep learning models can make a single prediction for each item, regardless of the number of components that make up the item.

### Multimodal Prediction

Multimodal data has been used for detecting outcomes such as sentiment (Deshpande et al., 2025) or fake news (Lin et al., 2024), but its application is new in the measurement field. Multimodal prediction approaches depend on how information from different modalities is represented and combined in a deep fusion model (Gao et al., 2020; W. Li, Peng, et al., 2023). One common strategy is early fusion, in which features from multiple modalities are concatenated at the input level and passed through a single predictive model (Baltrušaitis et al., 2018). While straightforward, early fusion assumes that all modalities are always present and equally informative. An alternative is late fusion, where separate modality-specific models learn independent representations or predictions that are subsequently combined (Ngiam et al., 2011), allowing greater robustness to missing data or heterogeneous inputs. Such flexible architecture is desirable in contexts where items vary in their inclusion of text, images, and metadata. We use a late fusion strategy in this article.

## Item Response Models

In the current study, the probability of examinee j with ability θ obtaining a score v out of possible score categories $r=1,2,\ldots ,m_{i}$ on polytomous item i is represented with the generalized partial credit model (GPCM; Muraki, 1992):

(1) $$p\left(x_{\mathrm{ij}}=v\right)=\frac{\exp[1.7a_{i}\left(\sum_{r=1}^{v}\theta_{j}-b_{\mathrm{ir}}\right)]}{\sum_{c=1}^{m_{i}}\exp[1.7a_{i}\sum_{r=1}^{c}\left(\theta_{j}-b_{\mathrm{ir}}\right)]},$$

where $x_{\mathrm{ij}}$ is the examinee’s response pattern, 1.7 is the scaling factor, $a_{i}$ is the discrimination, $b_{\mathrm{ir}}$ is the item step parameters, with a constraint that $\frac{1}{m_{i}-1}\sum_{r=2}^{m_{i}}b_{\mathrm{ir}}=b_{i1}$ . The c is an index used in the summation in the denominator. For dichotomous items with $m_{i}=2$ , the GPCM can be expressed as a 2-parameter logistic model (2PL; Birnbaum, 1968), which simplifies the probability of correct response to:

(2) $$p\left(x_{\mathrm{ij}}=2\right)=\frac{\exp[1.7a_{i}\left(\theta_{j}-b_{i1}\right)]}{1+\exp[1.7a_{i}\left(\theta_{j}-b_{i1}\right)]}.$$

For the rest of this article, we drop the i index for brevity. The parameter $b_{1}$ represents the overall item difficulty for both the 2PL and GPCM. The intercept $-1.7{\mathrm{ab}}_{1}$ is also meaningful for both models, as it represents the difficulty of the item when $\theta_{j}=0$ , which is similar to the Rasch definition of item difficulty.

## Method

### Data

We demonstrate the modeling approach using 40,965 items designed for English language arts (51.2%) and mathematics (48.8%) state summative assessments for students in Grades 3–8 and 11. Items were field-tested among students corresponding to the grade level of each item, then calibrated and vertically scaled. The target student sample size for each item was 2,000 ( M=2,902 , SD=2,296 ). Both good and poor-quality items previously accepted (85.3%) and rejected (14.7%) after field testing were included in this pool, respectively. Items were rejected for a variety of reasons, including poor psychometric quality or content-related concerns. We retained these items so the model could be trained and used on potentially poor-quality items, since item quality is unknown prior to field testing, thereby increasing the practical utility of our models. Distribution of θ was approximately N(0,1) when each subject and grade was weighted equally.

Data were structured in a hierarchical format where every item had one or more item component, including stimulus (i.e., passages or other context), prompts (i.e., stem), individual response options, and images (see Table A1 in Appendix). A “full text” component was created for each item, which was a concatenation of all text other than the stimuli. This component allowed the language model to consider the full context of the item at once. Items had an average of 4.9 ( SD=2.9 ) text components for a total of 202,410 across all items. All items had the full text and prompt text components. Images associated with stimuli and prompts were included in the data. Images that were part of response options or included as accessibility features were ignored. The data contained 5,387 unique images, shared across an average of 2.9 items. Only 15.2% of items had images, 81.4% of which were math. Items with images contained an average of 2.5 images (*SD* = 1.9).

Eleven item types were present, such as multiple choice, multiple select, short answer, and match-interaction (see Table A1 in Appendix). Dichotomous items were calibrated using the 2PL model, while the GPCM was used for polytomous items. Polytomous items had up to five score categories. A total of 10.5% of the items were polytomous, with 1.1% having four or five score categories. The item intercept $-1.7{\mathrm{ab}}_{1}$ , discrimination a , difficulty $b_{1}$ , and step thresholds $b_{2}$ to $b_{5}$ had a *M* (*SD*) of −0.9 (1.69), 0.89 (0.41), 0.74 (1.41), 0.61 (1.01), 1.03 (1.1), 0.94 (0.78), and 2.02 (0.68), respectively. We partitioned the items randomly into approximately 80% training, 10% validation, and 10% test data. Testlets where multiple items associated with the same stimulus were always included in the same data group.

### Target Item Parameters

We aimed to predict all seven parameters, $-1.7{\mathrm{ab}}_{1}$ , a , and $b_{1}$ to $b_{5}$ , as they each contribute to item characterization. However, we dropped $b_{1}$ and $b_{2}$ from the direct prediction for stability and consistency reasons. When discrimination a approaches 0, $b_{1}$ can become numerically unstable. The $b_{1}$ in our data ranged from −655 to 7,420, which are extreme outliers that could adversely affect the training. Therefore, instead of predicting $b_{1}$ directly, we derived $b_{1}$ from predicted $-1.7{\mathrm{ab}}_{1}$ and a , by $b_{1}=\frac{-1.7{\mathrm{ab}}_{1}}{-1.7a}$ . For polytomous items with m≥3 , we computed $b_{2}$ using the other predicted parameters by:

(3) $$b_{2}=b_{1}\left(m-1\right)-\sum_{r=3}^{m}b_{r}.$$

Deriving $b_{1}$ and $b_{2}$ rather than direct prediction ensured mathematical consistency among the parameters while reducing the influence of instability associated with extreme $b_{1}$ values. Prior to training, each item parameter was individually standardized to approximately N(0,1) to ensure they contribute equally to the loss function.

### Text Sub-Model

The text sub-model consists of the DeBERTaV3-large Transformer language model (He et al., 2021), followed by a 128-dimensional projection layer and a regression head. Data are processed entirely at the text component-level. Tokenized text is entered into DeBERTaV3, and the last hidden state of the [CLS] token is extracted and projected into a 128-dimensional hidden space, and used to predict the five aforementioned item parameters. Mathematically, the model is defined as follows. Suppose $x^{\mathrm{ik}}$ denotes the vector of input tokens for the k -th text component of item i . The textual representation is obtained by

(4) $$\begin{array}{c} h_{\mathrm{text}}^{\mathrm{ik}}=\mathrm{DeBERTaV}3\left(x_{\mathrm{text}}^{\mathrm{ik}}\right), \end{array}$$

where $h^{\mathrm{ik}}\in R^{128}$ is a hidden representation with 128 dimensions. The prediction layer is implemented as a feedforward neural network (FNN), given by

(5) $$\begin{array}{c} {\hat{y}}^{\mathrm{ik}}=W_{2}\cdot \mathrm{ReLU}\left(W_{1}h_{\mathrm{text}}^{\mathrm{ik}}+b_{1}\right)+b_{2}. \end{array}$$

here, ${\hat{y}}^{\mathrm{ik}}\in R^{d_{y}}$ denotes the predicted item parameters for item i , more specifically, as $d_{y}=5$ for $\left(-1.7a_{i}b_{i1},a_{i},b_{i3},b_{i4},b_{i5}\right)$ . The hidden layer is parameterized by the weight matrix $W_{1}\in R^{128\times 128}$ and bias vector $b_{1}\in R^{128}$ , while the output layer is defined by $W_{2}\in R^{d_{y}\times 128}$ and bias $b_{2}\in R^{d_{y}}$ . The activation function ReLU(·) is defined element-wise as ReLU(x)=max(0,x) .

### Image Sub-Model

The image sub-model consists of the DINOv3 vision Transformer (Siméoni et al., 2025), followed by a 128-dimensional projection layer and a regression head. Data are processed entirely at the image component level. Each PNG image pixel was typically represented by three numeric values corresponding to the RGB color channels. Images containing a fourth alpha (transparency) channel were converted by replacing transparent pixels with white. Consistent with the default approach for DINOv3, every image was resized to 224 × 224 pixels, where any rectangular images were stretched to a square (i.e., rather than cropped). Finally, images were passed through DINOv3. The last hidden state of the [CLS] token for each image was extracted and projected into a 128-dimensional hidden space, and used to predict $-1.7{\mathrm{ab}}_{1}$ , a , and $b_{3}$ . Parameters $b_{4}$ and $b_{5}$ were ignored for this model because of insufficient data. Mathematically, the model is defined as follows. Suppose $x^{\mathrm{ik}}$ denotes the vector of pixel values for the k -th image component of item i . The image representation is obtained by

(6) $$\begin{array}{c} h_{\mathrm{image}}^{\mathrm{ik}}=\mathrm{DINOv}3\left(x_{\mathrm{image}}^{\mathrm{ik}}\right), \end{array}$$

where $h^{\mathrm{ik}}\in R^{128}$ is a hidden representation with 128 dimensions. The prediction layer is implemented as a FNN, given by

(7) $$\begin{array}{c} {\hat{y}}^{\mathrm{ik}}=W_{2}\cdot \mathrm{Dropout}\left(\mathrm{ReLU}\left(W_{1}h_{\mathrm{image}}^{\mathrm{ik}}+b_{1}\right)\right)+b_{2}. \end{array}$$

here, ${\hat{y}}^{\mathrm{ik}}\in R^{d_{y}}$ denotes the predicted item parameters. The hidden layer is parameterized by the weight matrix $W_{1}\in R^{128\times 128}$ and bias vector $b_{1}\in R^{128}$ , while the output layer is defined by $W_{2}\in R^{d_{y}\times 128}$ and bias $b_{2}\in R^{d_{y}}$ . The dropout function (Srivastava et al., 2014), which is only activated during training, is included to regularize parameters and stabilize predictions.

### Multimodal Deep Fusion Model

**Figure 1.**
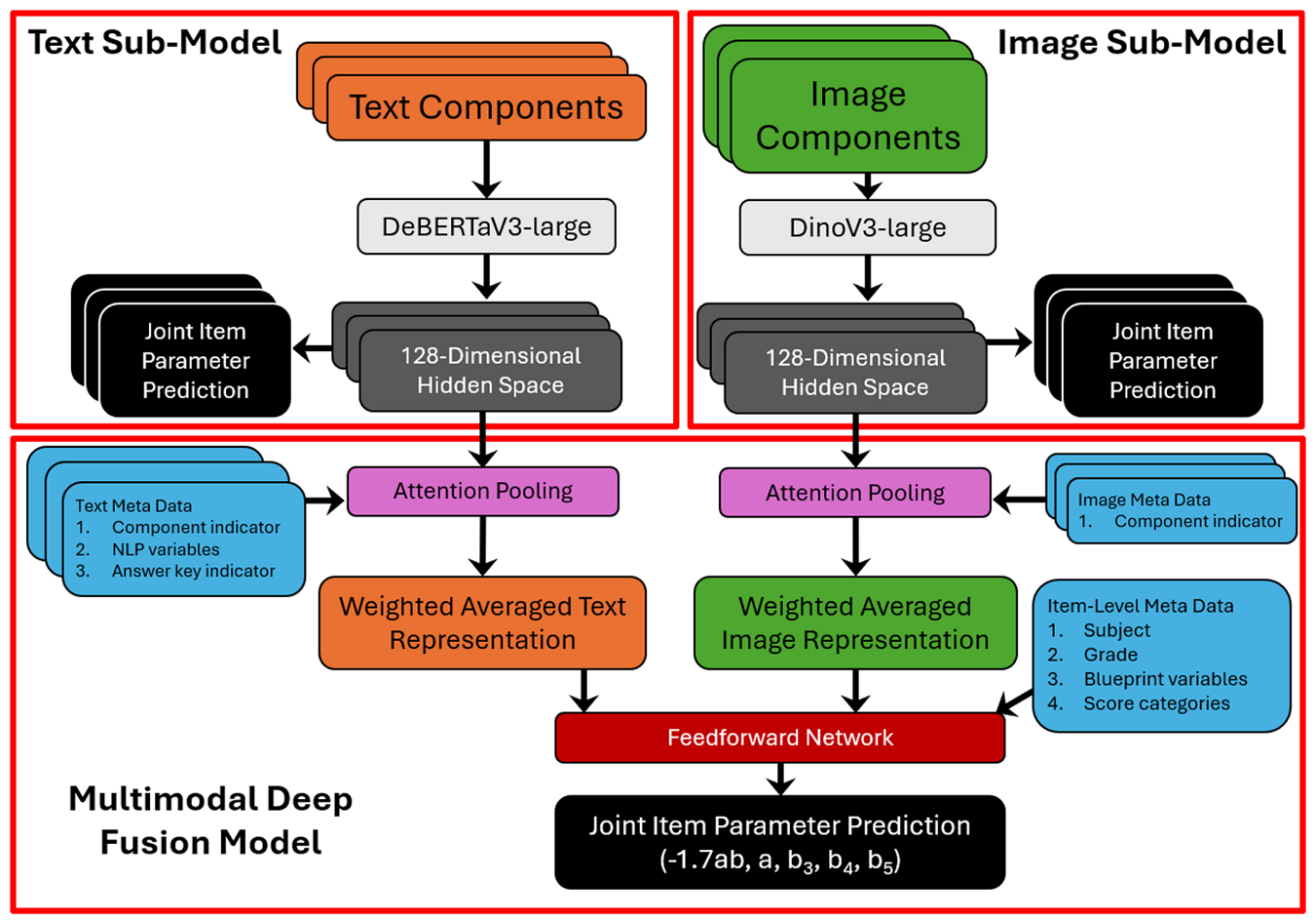
Multimodal deep fusion model diagram. *Note*. Text and image sub-models are fine-tuned separately, and then their outputs are later combined in the fusion model.

The multimodal deep fusion model integrates text and image–component level input into item-level representations through attention pooling, concatenates these with item-level metadata, projects the combined representation into a 128-dimensional hidden space, and applies a final regression head to predict all item parameters (see Figure 1). Initially, the text and image components are entered into each respective sub-model, which allowed us to convert each text and image component to a 128-dimensional representation. This output was subsequently combined with various metadata in the deep fusion model (Gao et al., 2020; W. Li, Peng, et al., 2023) using late fusion (Ngiam et al., 2011).

There were four groups of metadata: item-level variables, component indicators, answer key indicators, and text component-level natural language processing (NLP) variables. Item-level variables were mostly composed of dummy-coded blueprint-related variables, including the item subject, grade, item type, number of score categories, and depth of knowledge. Component indicators are dummy variables that indicate whether each image was included in the stimulus or prompt, and whether each text component was a stimulus, prompt, or options. Answer key indicators were also dummy variables that indicated whether each response option text component is a correct or incorrect answer. Answer key indicators were only relevant to items with options, which were multiple-choice or multiple-select items. NLP variables were calculated for each individual text component using the textstat package (Ward, 2025), including letter count, sentence count, characters per word, letters per word, sentences per word, words per sentence, syllables per word, Flesch reading ease, automated readability index, Dale-Chall readability score, Simple Measure of Gobbledygook (SMOG) index, and reading time. NLP variables were all standardized to approximately N(0,1) .

Overall, the deep fusion model is composed of three types of data: (1) image component–level data, including the 128-dimensional image space and image component indicators, (2) text-component-level data, including the 128-dimensional text space, text component indicators, answer key indicators, and NLP variables, and the (3) item-level metadata. The text and image component representations were aggregated into an item-level embedding using attention pooling (Er et al., 2016) with a hidden dimension of 64, applied separately to text and image features. We include text and image component indicator dummy variables to facilitate attention pooling. Image embeddings were imputed as 0 for items without images.

Mathematically, the attention pooling module is defined as follows. Let $m_{\mathrm{text}}^{\mathrm{ik}}\in R^{d_{\mathrm{text}}}$ denote the metadata for the k -th text component of item i , and let $m_{\mathrm{image}}^{\mathrm{ik}}\in {\left\{0,1\right\}}^{d_{\mathrm{image}}}$ denote the metadata for the k -th image component of item i . An attention pooling module is defined separately for each data type, where type∈{text,image} . The dimensionalities of the metadata vectors are set to $d_{\mathrm{text}}=27$ and $d_{\mathrm{image}}=2$ .

(8) $$\begin{array}{c} s_{\mathrm{type}}^{\mathrm{ik}}=W_{2}\cdot \mathrm{Tanh}\left(W_{1}\left[\begin{array}{c} h_{\mathrm{type}}^{\mathrm{ik}}\\ m_{\mathrm{type}}^{\mathrm{ik}} \end{array}\right]+b_{1}\right)+b_{2}, \end{array}$$

(9) $$\begin{array}{c} w_{\mathrm{type}}^{\mathrm{ik}}=\frac{\exp\left(s_{\mathrm{type}}^{\mathrm{ik}}\right)}{\sum_{v}^{N_{\mathrm{type}}^{i}}\exp\left(s_{\mathrm{type}}^{\mathrm{iv}}\right)}, \end{array}$$

(10) $$\begin{array}{c} p_{\mathrm{type}}^{i}=\sum_{k}^{N_{\mathrm{type}}^{i}}w_{\mathrm{type}}^{\mathrm{ik}}\left[\begin{array}{c} h_{\mathrm{type}}^{\mathrm{ik}}\\ m_{\mathrm{type}}^{\mathrm{ik}} \end{array}\right], \end{array}$$

where $N_{\mathrm{type}}^{i}$ denotes the number of text/image components associated with item i . The hidden layer is parameterized by the weight matrix $W_{1}\in R^{\left(128+d_{\mathrm{type}}\right)\times 64}$ and bias vector $b_{1}\in R^{64}$ , while the output layer is defined by $W_{2}\in R^{64\times 1}$ and bias $b_{2}\in R$ . The dimensionality of the pooled vectors is $p_{\mathrm{type}}^{i}\in R^{128+d_{\mathrm{type}}}$ .

Finally, the prediction layer is defined as follows

(11) $$\begin{array}{c} {\hat{y}}^{i}=W_{2}\cdot \mathrm{Dropout}\left(\mathrm{ReLU}\left(W_{1}\left[\begin{array}{c} p_{\mathrm{text}}^{i}\\ p_{\mathrm{image}}^{i}\\ d^{i} \end{array}\right]+b_{1}\right)\right)+b_{2}, \end{array}$$

where $d^{i}\in R^{d_{\mathrm{item}}}$ is a vector of item-level metadata for item i and the dimensionality of the item-level metadata vectors ( $d^{i}$ ) is 151. The hidden layer is parameterized by the weight matrix $W_{1}\in R^{128\times \left(256+d_{\mathrm{text}}+d_{\mathrm{image}}+d_{\mathrm{item}}\right)}$ and bias vector $b_{1}\in R^{128}$ , while the output layer is defined by $W_{2}\in R^{d_{y}\times 128}$ and bias $b_{2}\in R^{d_{y}}$ .

### Model Optimization

Text sub-model, image sub-model, and the fusion model were trained in Python using the pytorch library (Paszke et al., 2019). All parameters were predicted jointly within each model. Joint prediction can improve accuracy when target variables are correlated, as information shared across item parameters can be leveraged during training. In the present data, item parameters showed moderate correlations (mean pairwise absolute value of correlations was .42 ). Although 89.5% of items were dichotomous, the model was specified to output all parameters for every item, including three parameters only applicable to polytomous items. A masking technique was then applied during loss computation to ignore parameters that were not relevant for a given item, ensuring that those outputs did not contribute to the loss, gradient calculation, or parameter updates (Østmo et al., 2026; Xu et al., 2017).

We considered alternative strategies like (a) training separate models for each item parameter, (b) partitioning the models into English language arts and math, or (c) replicating each item once per item parameter, while including an indicator of the target parameter and predicting a single outcome. These approaches were computationally inefficient and inconvenient as they multiplied the training time and data volume. We also observed no definitive improvement to the loss during pilot testing.

DeBERTaV3 in the text sub-model was fine-tuned using a Low-Rank Adaptation (LoRA; Hu et al., 2022; LoRA rank = 50, LoRA alpha = 100, LoRA dropout = 0.05, dropout = 0.1, batch size = 8, max token length = 512, weight decay = .01, learning rate = 7e-5, epochs = 4). LoRA is a parameter-efficient fine-tuning method that freezes a pretrained model’s original weights and instead learns small low-rank matrices that modify selected layers. This dramatically reduces the number of trainable parameters, lowering memory, storage, and computational costs. DINOv3 in the image sub-model was also fine-tuned using a LoRA (LoRA rank = 50, LoRA Alpha = 100, LoRA dropout = 0.1, dropout = 0.2, batch size = 8, weight decay = 0.1, learning rate = 4e-5, epochs = 8). All hyperparameters were selected based on trial and error through minimizing the mean-squared-error (MSE) loss among the validation data.

Once the sub-models converted each text and image component to a 128-dimensional representation, they were entered into the deep fusion model as fixed values, along with the aforementioned metadata. Then, the deep fusion model was trained using joint prediction and masked loss (Dropout = 0.05, accumulation steps = 16, weight decay = 0.001, norm = 0, and learning rate for head = 5e-5, pool = 2e-5, and norm = 1e-5).

The reason for training the sub-models and fusion models separately was computational efficiency, especially because the NVIDIA A10G Tensor Core 24 GB graphics processor we used was relatively small. In all models, MSE loss was used for training. We retained the state with the lowest MSE validation loss out of all epochs.

### Model Comparison

Along with the full deep fusion model, multiple variations of a more parsimonious model were explored, focusing on whether complex techniques and data structures added unique value to the model. Inclusion and removal of seven features were explored: (1) text component-level hidden space, (2) image component–level hidden space, (3) item-level variables, (4) text component-level answer key indicators, (5) text component-level NLP variables, (6) attention pooling, and (7) restricting the text to only the prompt. Component indicators were always included, unless the entire text or image component was removed. Not all combinations of the model complexity were explored.

Model performance was evaluated using $R^{2}$ between the target label and predictions in the test data. The reported $R^{2}$ is defined as 1−MSE/variance , which accounts for bias in the predictions. This definition is more stringent than computing squared Pearson correlations. For a limited set of the best models, additional results are reported using root mean squared error (RMSE) and signed mean bias separately between exam subjects and items with images. We limited the range of $b_{1}$ to [−5,5] to reduce the impact of extreme outliers before calculating $R^{2}$ , RMSE, and bias.

## Results

### Vision Model Visualization

**Figure 2.**
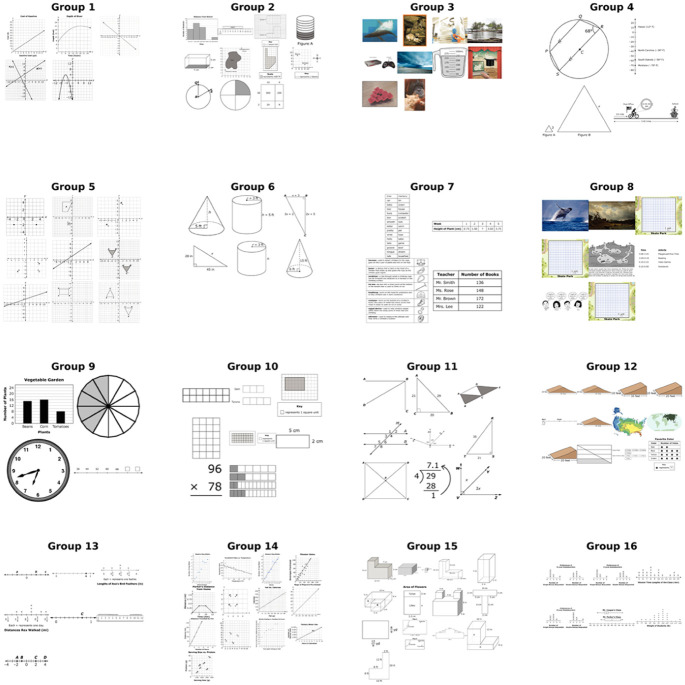
Item images categorized using *k*-means based on the 128-dimensional hidden space from DINOv3-large. *Note*. All training, validation, and test set data that we could publicly display are included. This figure shows the types of information that are captured by the AI vision model. *©2024 The Regents of the University of California. This work is provided by Smarter Balanced and openly licensed via CC BY-ND 4.0*

Prior to the main modeling results, we present two figures to help understand the information captured by the 128-dimensional DINOv3-large image embeddings. First, we applied a *k*-means clustering algorithm to partition the images into 16 semantically distinct categories. Representative images from each cluster were then displayed to illustrate the captured visual patterns. Training, validation, and test sets were combined for this procedure due to the limited number of images we could publicly display. The resulting clusters corresponded to visual structures such as line graphs, tables, geometric figures, colored diagrams, and photographic images (see Figure 2).

**Figure 3.**
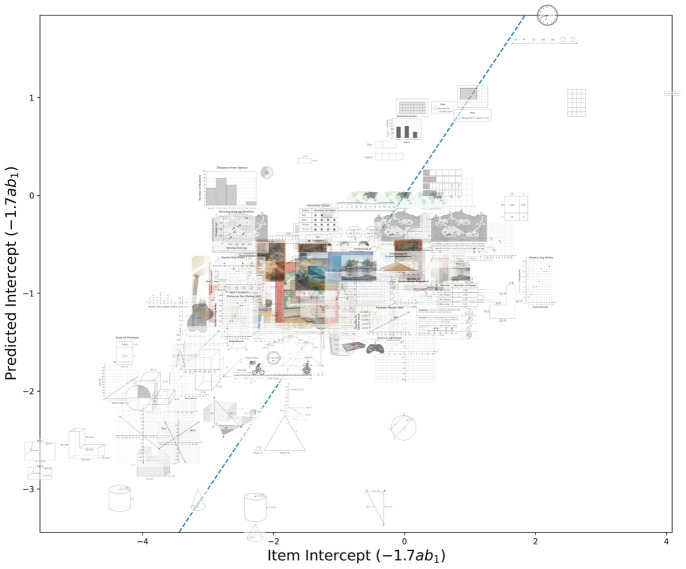
Scatterplot of the item intercepts and their predictions based on fine-tuned DINOv3-large item-image embeddings. *Note*. A limited set of training, validation, and test set data are included. Only image information was used for this prediction model. Higher values are easier items. *©2024 The Regents of the University of California. This work is provided by Smarter Balanced and openly licensed via CC BY-ND 4.0*

To present how DINOv3 linked the visual information to item parameters, we plotted the item intercept parameter (i.e., −1.7ab ) and its predicted value from the 128-dimensional image embeddings (i.e., see Figure 3). The prediction accuracy for the test set was $R^{2}=.25$ . Training, validation, and test sets were combined for this visualization. The figure shows the types of images that led to low (i.e., difficult) or high (i.e., easier) predicted intercept.

### Model Comparison Results

The rest of the reported results are based on the test data only. Table 1 shows the $R^{2}$ for the list of all model variants. Model 1 is the model with just mean pooling applied to the DeBERTaV3 text sub-model, while Model 2 adds attention pooling to this. Model 3 applied mean pooling to the DINOv3 image sub-model output, while Model 4 adds attention pooling to it. Model 5 includes all metadata and attention pooling features. Model 6 is the full model with all features (436 predictors and 75,337 parameters excluding DeBERTaV3 and DINOv3 parameters). To identify the features that are essential to the model performance, Models 7–13 remove parameters and predictors from the full model. To further explore parsimonious models, we tested using only the text of the prompt (i.e., Models 14–17).

**Table 1.** Model Comparisons Using $R^{2}$ .

								$R^{2}$						
Model	LM	VM	ILP	NLP	Key	AP	PO	$-1.7{\mathrm{ab}}_{1}$	a	$b_{1}$ ^a^	$b_{2}$ ^a^	$b_{3}$	$b_{4}$	$b_{5}$
1	√							.63	.44	.35	-.05	.65	.27	.68
2	√					√		.66	.45	.38	-.10	.64	.26	.67
3		√						.25	.19	.13	.09	.25		
4		√				√		.24	.19	.15	.03	.23		
5			√	√	√	√		.58	.40	.34	.28	.59	.26	.71
6^b^	√	√	√	√	√	√		.67	.46	.38	.28	.66	.29	.67
7		√	√	√	√	√		.58	.40	.34	.29	.60	.24	.69
8^b^	√		√	√	√	√		.67	.46	.38	.37	.66	.33	.74
9	√	√	√	√	√			.67	.45	.38	.38	.67	.31	.69
10	√		√	√	√			.67	.45	.37	.39	.67	.31	.75
11^b^	√		√	√		√		.67	.46	.38	.34	.66	.32	.62
12	√		√			√		.67	.46	.39	.22	.66	.28	.61
13	√		√					.67	.45	.37	.34	.66	.31	.69
14	√						√	.65	.44	.38	-.07	.64	.30	.73
15^b^	√		√				√	.67	.45	.39	.34	.66	.33	.74
16^b^	√		√	√			√	.67	.45	.39	.35	.66	.31	.71
17^b^	√	√	√				√	.67	.46	.39	.37	.66	.34	.74

*Note*. LM = language model projections, VM = vision model projections, ILP = item-level predictors, NLP = natural language processing predictors, Key = answer key indicators, AP = attention pooling, PO = prompt text component data only, $R^{2}=1-\mathrm{mean}\;\mathrm{squared}\;\mathrm{error}/\mathrm{variance}$ . Models 3 and 4 included only items with images. Model 6 is the full model with all 436 predictors and 75,337 parameters, excluding DeBERTaV3 and DINOv3 parameters. Model 14 is the simplest model with 131 predictors, 17,799 parameters, and uses only the prompt text component.

a Computed using the predicted parameters. ^b^Nearly tied for best-performing model.

The best average $R^{2}$ was .51 (i.e., weighted by the number of parameters), but six models were within 0.0025 of this (Models 6, 8, 11, 15, 16, and 17). The only two features that consistently contributed to increased $R^{2}$ were the DeBERTaV3 language model projections and item-level predictors. It was not surprising that item-level predictors improved the model because (1) subject can be used to rescale the item parameters between English language arts and math, while (2) grade level is a powerful predictor of item difficulty for these vertically scaled items. Including images, NLP variables, answer key indicators, or attention pooling as predictors did not always improve the prediction accuracy. Often, simplifying the model improved $R^{2}$ . Overall, every model was able to predict all item parameters to some degree, but there was a substantial amount of information redundancy and diminishing returns when adding more features.

To understand some of this information redundancy, we regressed the item intercept on item grade level ( $R^{2}=.21$ ) and compared it to a model that also included the image-only predictions from Model 3 ( $R^{2}=.30$ ). The incremental gain ( $\Delta R^{2}=.09$ ), relative to the $R^{2}$ from image-only predictions alone ( $R^{2}=.25$ ), indicates that the image-related signals captured by DINOv3 largely reflected grade-level differences. This is unsurprising, as assessment design systematically varies images across grades. The results highlight the challenge of training vision models to capture nuanced image features related to item parameters while ignoring easily recoverable metadata signals such as grade level.

To further examine the contribution of our best model and image features, we focus on Models 15 and 17 in the following section. Model 15 represents the most parsimonious specification among the six highest-performing models, whereas Model 17 extends it by incorporating the image component.

### Closer Look at Models 15 and 17

**Figure 4.**
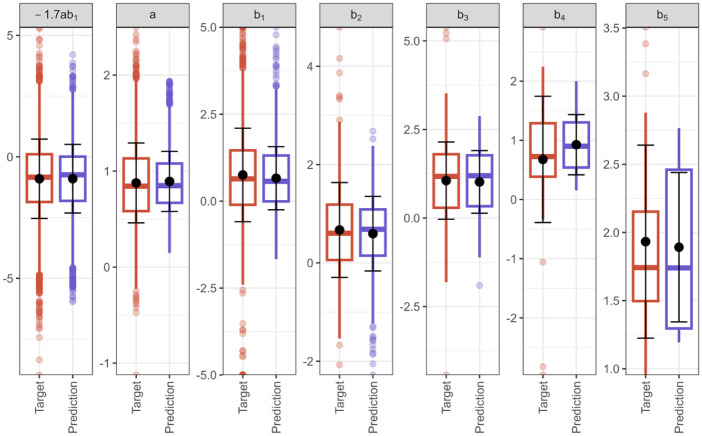
Boxplots of Model 15 target labels and predictions. *Note*. Mean and *SD* are shown in black. Model 15 was our best-performing parsimonious model, which included item-level predictors and language model projections of the item prompt text.

Boxplots of Model 15 output show a similar distribution shape, mean, and median as the target label, but predictions had a lower variance (see Figure 4). Regression to the mean is a typical phenomenon, but it is notable in this case because the model cannot be used for detecting poor-quality items with low discrimination or that are too difficult or easy. Predictions of a ranged from 0.15 to 1.9, despite having 0.9% negative values in the original data. The $-1.7{\mathrm{ab}}_{1}$ ranged from −9.0 to 5.3 in the data, but predictions were limited between −6.2 and 4.2.

Comparisons of Model 15 and 17 RMSE show that the inclusion of the image components did not change the prediction accuracy substantially (see Table 2). The most notable change was a decrease in $-1.7{\mathrm{ab}}_{1}$ RMSE by 0.02 for both subjects.

**Table 2.** RMSE for Models 15 and 17.

Subject	Items with Images only	Model	N items	RMSE						
				$-1.7{\mathrm{ab}}_{1}$	a	$b_{1}$ ^a^	$b_{2}$ ^a^	$b_{3}$	$b_{4}$	$b_{5}$
ELA		15	2,119	0.82	0.30	1.12	0.64	0.47	0.20	0.34
		17	2,119	0.82	0.30	1.12	0.60	0.47	0.20	0.35
	Yes	15	102	0.69	0.22	0.52	0.60	0.38	0.17	0.21
	Yes	17	102	0.67	0.22	0.53	0.56	0.38	0.18	0.20
Math		15	1,969	1.05	0.31	0.98	0.95	0.82	1.29	0.49
		17	1,969	1.05	0.31	0.98	0.95	0.82	1.29	0.43
	Yes	15	518	1.10	0.28	1.05	0.90	0.66	0.89	
	Yes	17	518	1.08	0.28	1.06	0.89	0.66	0.89	
All		15	4,088	0.94	0.31	1.05	0.78	0.64	0.86	0.35
		17	4,088	0.94	0.31	1.05	0.77	0.64	0.86	0.36

*Note*. RMSE = root mean squared error, ELA = English language arts. Model 15 was our best-performing parsimonious model, which included item-level predictors and language model projections of the item prompt text. Model 17 included image components in addition to the features in Model 15.

a Computed using the predicted parameters.

Comparisons of Model 15 and 17 mean signed bias show that the inclusion of the image components did not change the predictions substantially (see Table 3). The largest notable change was $-1.7{\mathrm{ab}}_{1}$ for math items that had images (bias reduced from 0.09 to 0.04).

**Table 3.** Bias for Models 15 and 17.

Subject	Items with Images only	Model	N items	Mean signed bias						
				$-1.7{\mathrm{ab}}_{1}$	a	$b_{1}$ ^a^	$b_{2}$ ^a^	$b_{3}$	$b_{4}$	$b_{5}$
ELA		15	2,119	-0.03	-0.01	0.16	0.07	0.05	-0.02	0.02
		17	2,119	-0.04	-0.01	0.17	0.10	0.03	0.00	0.04
	Yes	15	102	0.13	0.01	-0.04	0.10	-0.00	-0.03	-0.09
	Yes	17	102	0.10	0.02	-0.02	0.07	-0.01	-0.03	-0.05
Math		15	1,969	0.03	-0.02	0.02	0.08	0.02	-0.55	0.49
		17	1,969	0.01	-0.02	0.03	0.11	0.02	-0.53	0.43
	Yes	15	518	0.09	-0.03	-0.03	-0.02	-0.12	-0.27	
	Yes	17	518	0.04	-0.02	0.01	0.08	-0.11	-0.26	
All		15	4,088	-0.00	-0.02	0.09	0.08	0.04	-0.25	0.04
		17	4,088	-0.02	-0.01	0.10	0.11	0.03	-0.23	0.05

*Note*. ELA = English language arts. Model 15 was our best-performing parsimonious model, which included item-level predictors and language model projections of the item prompt text. Model 17 included image components in addition to the features in Model 15.

a Computed using the predicted parameters.

Model 15 performance in handling each item type was examined (see Table 4). The $R^{2}$ was negative for a few cases with a small sample size, such as hot-text-question-orderable ( n=5 ) or match-interaction ( n=55 ) English language arts items. Although multiple-choice items were the most common type, prediction accuracy was relatively low, with $-1.7{\mathrm{ab}}_{1}\;R^{2}$ values of .37 and .48 for English language arts and math, respectively. Predictions for multiple-choice items could improve if response option text and answer key indicators could be leveraged reliably. Interestingly, prediction accuracy was relatively high for purely mathematics items like equations ( $-1.7{\mathrm{ab}}_{1}\;R^{2}=.70$ ), despite relying on a language model not specifically designed to understand math.

**Table 4.** Model 15 Prediction Accuracy by Subject and Item Type.

			$R^{2}$			RMSE			Mean signed bias		
Subject	Item type	*N* items	$-1.7{\mathrm{ab}}_{1}$	a	$b_{1}$ ^a^	$-1.7{\mathrm{ab}}_{1}$	a	$b_{1}$ ^a^	$-1.7{\mathrm{ab}}_{1}$	a	$b_{1}$ ^a^
ELA	EBSR	198	.28	.05	.10	0.94	0.36	1.28	-0.11	-0.03	0.20
	HTQO	5	-.12	-1.24	-.04	1.37	0.33	1.64	-1.30	-0.27	1.29
	HTQS	295	.53	.18	.29	0.88	0.31	1.19	0.03	-0.03	0.20
	MC	925	.37	.13	.13	0.84	0.33	1.22	-0.03	0.01	0.16
	MI	55	.53	-.09	.13	1.16	0.36	1.47	0.23	-0.08	-0.05
	MS	393	.44	.10	.29	0.72	0.25	0.93	-0.02	-0.02	0.19
	SA	200	.55	.32	.39	0.58	0.17	0.41	-0.06	0.00	0.04
	WER	48	.72	.45	.72	0.43	0.21	0.28	-0.09	-0.02	0.07
Math	EQ	785	.70	.33	.68	1.23	0.32	0.62	-0.01	-0.01	0.01
	GI	222	.63	.34	.65	1.12	0.34	0.73	0.18	-0.05	0.03
	MC	472	.48	.24	.25	0.74	0.27	1.31	-0.04	-0.02	0.07
	MI	235	.58	.44	.33	1.01	0.32	1.34	0.01	-0.01	0.04
	MS	145	.40	.32	.14	0.90	0.31	1.20	0.19	-0.03	-0.02
	SA	72	.51	.32	.57	0.98	0.28	0.46	0.12	-0.05	-0.02
	TI	38	.83	.34	.81	0.91	0.27	0.51	0.06	-0.01	-0.05

*Note*. ELA = English language arts, EBSR = evidence-based selected response, EQ = equation, HTQO = hot-text-question-orderable, HTQS = hot-text-question-selectable, GI = grid item, MC = multiple choice, MI = match-interaction, MS = multiple select, SA = short answer, TI = table interaction, WER = writing extended response, $R^{2}=1-\mathrm{mean}\;\mathrm{squared}\;\mathrm{error}/\mathrm{variance}$ , RMSE = root mean squared error. Model 15 was our best-performing parsimonious model, which included item-level predictors and language model projections of the item prompt text.

a Computed using the predicted parameters.

## Discussion

To our knowledge, this is the first study to predict item parameters using images through an artificial intelligence (AI)-based vision model or multimodal model. A key contribution is the flexibility of the proposed approach relative to prior work (AlKhuzaey et al., 2024; Benedetto et al., 2023; Peters et al., 2025). By including mechanisms that can capture nearly all information present in the item data, the model can accommodate a wide range of item formats, extending its applicability across diverse testing programs. A single model successfully accommodated both exam subjects, all 11 item types, and all item parameters, eliminating the need for multiple specialized models. Joint parameter prediction along with a masked loss strategy substantially reduced the amount of processed data and training time. More broadly, the general framework of combining item components through attention pooling and fusing modality-specific models has applications beyond item parameter prediction. Predicting item parameters creates opportunities to revise and improve items prior to field testing, potentially reducing the number of rejected items. These capabilities are especially powerful when paired with automated item generation, where evaluating item quality is becoming more challenging than generating new items.

Despite these advantages, the models were unable to fully leverage all of the information available in the data. Although components such as image embeddings, attention pooling, and answer key indicators showed potential, oftentimes, prediction accuracy decreased as features were added. Results suggest the model’s difficulty learning stable and generalizable patterns from the expanded feature space during the training. This may reflect excessive model complexity relative to sample size, redundancy across predictors, or limitations in training and fine-tuning that hindered the separation of real signal from noise.

Consequently, a more parsimonious model performed about the same or better than more complex variants. Out of the six best-performing models, Model 15 was the most parsimonious, which achieved overall $R^{2}$ values of .67, .45, .39, .34, .66, .33, and .74 for parameters $-1.7{\mathrm{ab}}_{1}$ , a , and $b_{1}$ through $b_{5}$ , respectively. Notably, this model relied on a relatively simple architecture using item-level predictors and language model projections of the item prompt text. Its performance suggests that, under current training conditions, simpler models may be more effective for item parameter prediction. These results do not imply that the item prompt alone captures all relevant item information. Rather, they indicate limitations in current training methods for exploiting all available data.

These training shortcomings warrant the exploration of alternative regularization techniques, which are strategies that enhance model generalizability. In the current paper, we used regularization methods such as dropout and weight decay, but there are a broader range of techniques that may further reduce overfitting (Tian & Zhang, 2022). For example, image data augmentation techniques, including random rotations and cropping, could be applied during training to encourage the vision model to learn more invariant visual representations rather than memorizing specific images.

Model performance may also be improved through better integration of visual and textual information. In order for image and text data to complement each other, both alignment and fusion are essential (S. Li & Tang, 2025). Semantic correspondences between modalities should be established through alignment, then fused together to make unified predictions. Recently, “multimodal large language models” trained specifically on both text and images have emerged (e.g., J. Han, Gong, et al., 2025; Peng et al., 2023). These new models may enhance cross-modal interaction while simplifying model design compared to the complex fusion strategies employed in this study.

## Appendix

**Table A1.** Distribution of Item Components by Modality, Subject, and Item Type.

Modality	Subject	Component	Item types	n
Image	ELA	Prompt	HTQS, MC, MS	230
		Stimulus	EBSR, HTQS,MC,MI, MS, SA	4,532
	Math	Prompt	EQ, GI, HTQS,MC,MI,MS,SA,TI	9,077
		Stimulus	EQ, GI,MC,MI,MS,SA,TI	1,848
Text	ELA	Full text	EBSR, HTQS,MC,MI,MS,SA,WER	20,957
		Prompt	EBSR, HTQO,HTQS,MC,MI,MS,SA,WER	20,957
		HTQS Prompt	HTQS	3,087
		EBSR prompt A	EBSR	1,887
		EBSR prompt B	EBSR	1,887
		HTQO	HTQO	236
		Option	EBSR, MC,MS	72,522
		Stimulus	EBSR, HTQO,HTQS,MC,MI,MS,SA,WER	13,383
		Stimulus (audio)	EBSR, MC,MI,MS	836
	Math	Full text	EBSR, EQ,GI,HTQS,MC,MI,MS,SA,TI	20,008
		Prompt	EBSR, EQ,GI,HTQS,MC,MI,MS,SA,TI	20,008
		HTQS Prompt	HTQS	1
		EBSR prompt A	EBSR	5
		EBSR prompt B	EBSR	5
		Option	EBSR, MC,MS	24,991
		Stimulus	EBSR, EQ,GI,MC,MI,MS,SA,TI	1,640

*Note*. ELA = English language arts, EBSR = evidence-based selected response, EQ = equation, HTQO = hot-text-question-orderable, HTQS = hot-text-question-selectable, GI = grid item, MC = multiple choice, MI = match-interaction, MS = multiple select, SA = short answer, TI = table interaction, WER = writing extended response. Data contained a total of 40,965 items, where each item was structured in a hierarchical manner, made up of zero or more image and text components.

## References

[bibr1-00131644261460779] AlKhuzaey S. Grasso F. Payne T. R. Tamma V. (2024). Text-based question difficulty prediction: A systematic review of automatic approaches. International Journal of Artificial Intelligence in Education, 34(3), 862–914.

[bibr2-00131644261460779] Baltrušaitis T. Ahuja C. Morency L.-P. (2018). Multimodal machine learning: A survey and taxonomy. IEEE Transactions on Pattern Analysis and Machine Intelligence, 41(2), 423–443. 29994351 10.1109/TPAMI.2018.2798607

[bibr3-00131644261460779] Baylari A. Montazer G. A. (2009). Design a personalized e-learning system based on item response theory and artificial neural network approach. Expert Systems with Applications, 36(4), 8013–8021.

[bibr4-00131644261460779] Benedetto L. (2023). A quantitative study of NLP approaches to question difficulty estimation. In International conference on artificial intelligence in education (pp. 428–434). Springer.

[bibr5-00131644261460779] Benedetto L. Cappelli A. Turrin R. Cremonesi P. (2020). R2DE: A NLP approach to estimating IRT parameters of newly generated questions. In Proceedings of the tenth international conference on learning analytics & knowledge (pp. 412–421). Association for Computing Machinery.

[bibr6-00131644261460779] Benedetto L. Cremonesi P. Caines A. Buttery P. Cappelli A. Giussani A. Turrin R. (2023). A survey on recent approaches to question difficulty estimation from text. ACM Computing Surveys, 55(9), 1–37.

[bibr7-00131644261460779] Birnbaum A. (1968). Some latent trait models and their use in inferring an examinee’s ability. In Lord F. M. Novick M. R. (Eds.), Statistical theories of mental test scores (pp. 397–479). Addison-Wesley.

[bibr8-00131644261460779] Circi R. Hicks J. Sikali E. (2023). Automatic item generation: Foundations and machine learning-based approaches for assessments. Frontiers in Education, 8, Article 858273.

[bibr9-00131644261460779] Crocker L. M. Algina J. (1986). Introduction to classical and modern test theory. Holt, Rinehart, and Winston.

[bibr10-00131644261460779] de Ayala R. J. (2009). The theory and practice of item response theory. Guilford Press.

[bibr11-00131644261460779] Deshpande U. U. Shanbhag S. Sukhasare A. Dixit M. M. Patil R. Sangani S. Srinivasaiah S. H. Goudar S. Managuli M. (2025). Multimodal sentiment analysis using image and text fusion for emotion detection. Discover Computing, 28(1), 1–24.

[bibr12-00131644261460779] Devlin J. Chang M. W. Lee K. Toutanova K. (2019). Bert: Pre-training of deep bidirectional transformers for language understanding. In NAACL HLT 2019 - 2019 Conference of the North American Chapter of the Association for Computational Linguistics: Human Language Technologies - Proceedings of the Conference, volume 1 (pp. 4171–4186). Association for Computational Linguistics.

[bibr13-00131644261460779] El Masri Y. H. Ferrara S. Foltz P. W. Baird J.-A. (2017). Predicting item difficulty of science national curriculum tests: The case of key stage 2 assessments. The Curriculum Journal, 28(1), 59–82.

[bibr14-00131644261460779] Er M. J. Zhang Y. Wang N. Pratama M. (2016). Attention pooling-based convolutional neural network for sentence modelling. Information Sciences, 373, 388–403.

[bibr15-00131644261460779] Gao J. Li P. Chen Z. Zhang J. (2020). A survey on deep learning for multimodal data fusion. Neural Computation, 32(5), 829–864. 32186998 10.1162/neco_a_01273

[bibr16-00131644261460779] Haladyna T. M. Downing S. M. Rodriguez M. C. (2002). A review of multiple-choice item-writing guidelines for classroom assessment. Applied Measurement in Education, 15(3), 309–333.

[bibr17-00131644261460779] Han J. Gong K. Zhang Y. Wang J. Zhang K. Lin D. Qiao Y. Gao P. Yue X. (2025). Onellm: One framework to align all modalities with language. arXiv preprint arXiv:2312.03700.

[bibr18-00131644261460779] Han S. Rijmen F. Boykin A. A. Lottridge S. (2025). Leveraging fine-tuned large language models in item parameter prediction. In Proceedings of the Artificial Intelligence in Measurement and Education Conference (AIME-Con): Full Papers (pp. 250–264). National Council on Measurement in Education.

[bibr19-00131644261460779] He P. Gao J. Chen W. (2021). Debertav3: Improving deberta using electra-style pre-training with gradient-disentangled embedding sharing. arXiv preprint arXiv:2111.09543.

[bibr20-00131644261460779] Hillsley K. (2025). Question format is the best predictor of item discrimination: A multivariable analysis. Journal of Microbiology and Biology Education, 26(3), Article e0020525. 10.1128/jmbe.00205-25 PMC12687604 41196037

[bibr21-00131644261460779] Hsu F.-Y. Lee H.-M. Chang T.-H. Sung Y.-T. (2018). Automated estimation of item difficulty for multiple-choice tests: An application of word embedding techniques. Information Processing & Management, 54(6), 969–984.

[bibr22-00131644261460779] Hu E. J. Shen Y. Wallis P. Allen-Zhu Z. Li Y. Wang S. Wang L. Chen W. (2022). Lora: Low-rank adaptation of large language models. ICLR, 1(2), 3.

[bibr23-00131644261460779] Khan A. Rauf Z. Sohail A. Khan A. R. Asif H. Asif A. Farooq U. (2023). A survey of the vision transformers and their CNN-transformer based variants. Artificial Intelligence Review, 56(Suppl. 3), 2917–2970.

[bibr24-00131644261460779] Kim G. Hong T. Yim M. Nam J. Park J. Yim J. Hwang W. Yun S. Han D. Park S. (2022). OCR-Free document understanding transformer. In European Conference on Computer Vision (pp. 498–517). Springer.

[bibr25-00131644261460779] Li J. Li D. Savarese S. Hoi S. (2023). Blip-2: Bootstrapping language-image pre-training with frozen image encoders and large language models. In International conference on machine learning (pp. 19730–19742). PMLR.

[bibr26-00131644261460779] Li J. Li D. Xiong C. Hoi S. (2022). Blip: Bootstrapping language-image pre-training for unified vision-language understanding and generation. In International conference on machine learning (pp. 12888–12900). PMLR.

[bibr27-00131644261460779] Li M. Jiao H. Zhou T. Zhang N. Peters S. Lissitz R. W. (2025). Item difficulty modeling using fine-tuned small and large language models. Educational and Psychological Measurement, 85(6), 1065–1090. 40630261 10.1177/00131644251344973 PMC12230038

[bibr28-00131644261460779] Li S. Tang H. (2025). Multimodal alignment and fusion: A survey. arXiv preprint arXiv:2411.17040.

[bibr29-00131644261460779] Li W. Peng Y. Zhang M. Ding L. Hu H. Shen L. (2023). Deep model fusion: A survey. arXiv preprint arXiv:2309.15698. 10.1109/TNNLS.2025.3628666 41289107

[bibr30-00131644261460779] Lin S.-Y. Chen Y.-C. Chang Y.-H. Lo S.-H. Chao K.-M. (2024). Text–image multimodal fusion model for enhanced fake news detection. Science Progress, 107(4), 00368504241292685. 10.1177/00368504241292685 PMC11500224 39440371

[bibr31-00131644261460779] Liu Y. Ott M. Goyal N. Du J. Joshi M. Chen D. Levy O. Lewis M. Zettlemoyer L. Stoyanov V. (2019). Roberta: A robustly optimized Bert pretraining approach. arXiv preprint arXiv:1907.11692.

[bibr32-00131644261460779] Maeda H. (2025). Field-testing multiple-choice questions with AI examinees: English grammar items. Educational and Psychological Measurement, 85(2), 221–244. 39554772 10.1177/00131644241281053 PMC11562880

[bibr33-00131644261460779] Maeda H. Lu Y. (2025). Finding words associated with DIF: Predicting differential item functioning using LLMs and explainable AI. Journal of Educational Measurement, 62(4), 883–906.

[bibr34-00131644261460779] Mayer R. E. (2002). Multimedia learning. Psychology of Learning and Motivation, 41, 85–139.

[bibr35-00131644261460779] Muraki E. (1992). A generalized partial credit model: Application of an EM algorithm. ETS Research Report Series, 1992(1), i–30.

[bibr36-00131644261460779] Ngiam J. Khosla A. Kim M. Nam J. Lee H. Ng A. Y. (2011). Multimodal deep learning. ICML, 11, 689–696.

[bibr37-00131644261460779] Østmo E. A. Radiya K. Wickstrøm K. K. Kampffmeyer M. Mikalsen K. Ø. Jenssen R. (2026). Liver, vessel, and tumor segmentation from partially labeled CT and multi-label masked learning. In Northern Lights Deep Learning Conference 2026 (pp. 415–427). PMLR.

[bibr38-00131644261460779] Oquab M. Darcet T. Moutakanni T. Vo H. Szafraniec M. Khalidov V. Fernandez P. Haziza D. Massa F. El-Nouby A. Assran M. Ballas N. Galuba W. Howes R. Huang P.-Y. Li S.-W. Misra I. Rabbat M. Sharma V. ...Bojanowski P. (2023). Dinov2: Learning robust visual features without supervision. arXiv preprint arXiv:2304.07193.

[bibr39-00131644261460779] Paivio A. (1990). Mental representations: A dual coding approach. Oxford university press.

[bibr40-00131644261460779] Paszke A. Gross S. Massa F. Lerer A. Bradbury J. Chanan G. Killeen T. Lin Z. Gimelshein N. Antiga L. Desmaison A. Köpf A. Yang E. DeVito Z. Raison M. Tejani A. Chilamkurthy S. Steiner B. Fang L. Chintala S. (2019). PyTorch: An imperative style, high-performance deep learning library. In H. Wallach, H. Larochelle, A. Beygelzimer, F. d'Alché-Buc, E. Fox, & R. Garnett (Eds.), Advances in Neural Information Processing Systems 32 (pp. 8024–8035). Curran Associates, Inc.

[bibr41-00131644261460779] Peng Z. Wang W. Dong L. Hao Y. Huang S. Ma S. Wei F. (2023). Kosmos-2: Grounding multimodal large language models to the world. arXiv preprint arXiv:2306.14824.

[bibr42-00131644261460779] Peters S. Zhang N. Jiao H. Li M. Zhou T. Lissitz R. (2025). Text-based approaches to item difficulty modeling in large-scale assessments: A systematic review. arXiv preprint arXiv:2509.23486.

[bibr43-00131644261460779] Radford A. Kim J. W. Hallacy C. Ramesh A. Goh G. Agarwal S. Sastry G. Askell A. Mishkin P. Clark J. Krueger G. Sutskever I. (2021). Learning transferable visual models from natural language supervision. In Meila M. Zhang T. (Eds.), Proceedings of the 38th International Conference on Machine Learning (Vol. 139, pp. 8748–8763). PMLR.

[bibr44-00131644261460779] Scarlatos A. Fernandez N. Ormerod C. Lottridge S. Lan A. (2025). Smart: Simulated students aligned with item response theory for question difficulty prediction. In Proceedings of the 2025 Conference on Empirical Methods in Natural Language Processing (pp. 25082–25105). Association for Computational Linguistics.

[bibr45-00131644261460779] Schnotz W. (2005). An integrated model of text and picture comprehension. The Cambridge Handbook of Multimedia Learning, 49(2005), 69.

[bibr46-00131644261460779] Siméoni O. Vo H. V. Seitzer M. Baldassarre F. Oquab M. Jose C. Khalidov V. Szafraniec M. Yi S. Ramamonjisoa M. Massa F. Haziza D. Wehrstedt L. Wang J. Darcet T. Moutakanni T. Sentana L. Roberts C. Vedaldi A. ...Bojanowski P. (2025). Dinov3. arXiv preprint arXiv:2508.10104.

[bibr47-00131644261460779] Smith R. (2007). An overview of the tesseract OCR engine. In Ninth international conference on document analysis and recognition (ICDAR 2007) (Volume 2, pp. 629–633). IEEE.

[bibr48-00131644261460779] Srivastava N. Hinton G. Krizhevsky A. Sutskever I. Salakhutdinov R. (2014). Dropout: A simple way to prevent neural networks from overfitting. Journal of Machine Learning Research, 15(1), 1929–1958.

[bibr49-00131644261460779] Stanger-Hall K. F. (2012). Multiple-choice exams: An obstacle for higher-level thinking in introductory science classes. CBE—Life Sciences Education, 11(3), 294–306. 22949426 10.1187/cbe.11-11-0100 PMC3433302

[bibr50-00131644261460779] Susanti Y. Tokunaga T. Nishikawa H. Obari H. (2017). Controlling item difficulty for automatic vocabulary question generation. Research and Practice in Technology Enhanced Learning, 12(1), 25. 30595730 10.1186/s41039-017-0065-5 PMC6294197

[bibr51-00131644261460779] Tian Y. Zhang Y. (2022). A comprehensive survey on regularization strategies in machine learning. Information Fusion, 80, 146–166.

[bibr52-00131644261460779] Ulitzsch E. Belov D. Luedtke O. Robitzsch A. (2025). Using item parameter predictions for reducing calibration sample requirements—A case study based on a high-stakes admission test. Journal of Educational Measurement, 63(1), Article e12426.

[bibr53-00131644261460779] Vaswani A. Shazeer N. Parmar N. Uszkoreit J. Jones L. Gomez A. N. Kaiser L. Polosukhin I. (2017). Attention is all you need. arXiv preprint arXiv:1706.03762.

[bibr54-00131644261460779] Ward A. (2025). Textstat (Version 1.0.0a0) [Computer software]. PyPI. https://pypi.org/project/textstat/1.0.0a0/

[bibr55-00131644261460779] Wauters K. Desmet P. Van Den Noortgate W. (2012). Item difficulty estimation: An auspicious collaboration between data and judgment. Computers & Education, 58(4), 1183–1193.

[bibr56-00131644261460779] Wu H.-K. Kuo C.-Y. Jen T.-H. Hsu Y.-S. (2015). What makes an item more difficult? Effects of modality and type of visual information in a computer-based assessment of scientific inquiry abilities. Computers & Education, 85, 35–48.

[bibr57-00131644261460779] Xu J. Wu S. Zhu S. Guo H. Wang H. Yang Q. (2017). Masked loss residual convolutional neural network for facial keypoint detection. In Proceedings of the 10th EAI International Conference on Mobile Multimedia Communications (pp. 234–239). Institute for Computer Sciences, Social-Informatics and Telecommunications Engineering.

[bibr58-00131644261460779] Yi X. Sun J. Wu X. (2024). Novel feature-based difficulty prediction method for mathematics items using XGBoost-based SHAP model. Mathematics, 12(10), 1455.

